# Avoiding Weight Gain in Cardiometabolic Disease: A Systematic Review

**DOI:** 10.1155/2014/358919

**Published:** 2014-12-28

**Authors:** Nisa M. Maruthur, Kimberly Gudzune, Susan Hutfless, Oluwakemi A. Fawole, Renee F. Wilson, Brandyn D. Lau, Cheryl A. M. Anderson, Sara N. Bleich, Jodi Segal

**Affiliations:** ^1^Division of General Internal Medicine, Johns Hopkins University School of Medicine, Baltimore, MD 21287, USA; ^2^Welch Center for Prevention, Epidemiology, and Clinical Research, Baltimore, MD 21287, USA; ^3^Department of Epidemiology, Johns Hopkins University School of Medicine, Baltimore, MD 21287, USA; ^4^Division of Gastroenterology, Johns Hopkins University School of Medicine, Baltimore, MD 21287, USA; ^5^Department of Health Policy and Management, Johns Hopkins University Bloomberg School of Public Health, Baltimore, MD 21287, USA; ^6^Division of Acute Surgery, Department of Surgery, Johns Hopkins University School of Medicine, Baltimore, MD 21287, USA; ^7^Division of Health Sciences Informatics, Johns Hopkins University School of Medicine, Baltimore, MD 21287, USA; ^8^Armstrong Institute for Patient Safety and Quality, Johns Hopkins Medicine, Baltimore, MD 21287, USA; ^9^Department of Family and Preventive Medicine, University of California at San Diego, San Diego, CA 92093, USA

## Abstract

Patients with cardiometabolic disease are at higher risk for obesity-related adverse effects. Even without weight loss, weight maintenance may be beneficial. We performed a systematic review to identify the effect of nonweight loss-focused lifestyle interventions in adults with cardiometabolic disease. We searched MEDLINE, Embase, and the Cochrane Central Register of Controlled Trials to identify comparative studies of lifestyle interventions (self-management, diet, exercise, or their combination) without a weight loss focus in adults with or at risk for diabetes and cardiovascular disease. Weight, BMI, and waist circumference at ≥12 months were the primary outcomes. Of 24,870 citations, we included 12 trials (self-management, *n* = 2; diet, *n* = 2; exercise, *n* = 2; combination, *n* = 6) studying 4,206 participants. Self-management plus physical activity ± diet versus minimal/no intervention avoided meaningful weight (−0.65 to −1.3 kg) and BMI (−0.4 to −0.7 kg/m^2^) increases. Self-management and/or physical activity prevented meaningful waist circumference increases versus control (−2 to −4 cm). In patients with cardiometabolic disease, self-management plus exercise may prevent weight and BMI increases and self-management and/or exercise may prevent waist circumference increases versus minimal/no intervention. Future studies should confirm these findings and evaluate additional risk factors and clinical outcomes.

## 1. Introduction

Patients with or at risk for diabetes and cardiovascular disease (CVD) are at particular risk for adverse effects of obesity. Obesity increases blood pressure [1] and causes insulin resistance [2] both of which contribute to development of hypertension [3], CVD [4–6], diabetes [7], and their complications including congestive heart failure, recurrent CVD events and death [4, 6], diabetic nephropathy, and diabetic retinopathy [8]. Most lifestyle interventions addressing weight in these populations focus on weight loss or maintenance of weight loss [9–11]. These weight loss interventions tend to be quite intensive to produce modest weight reductions but typically result in improved outcomes downstream of weight including reductions in diabetes risk, abnormal glucose homeostasis, low density lipoprotein (LDL) cholesterol, and blood pressure [9–12]. In contrast, weight neutral interventions may aim to prevent weight gain or emphasize lifestyle changes to maintain current weight. Trials have demonstrated that weight neutral interventions can improve metabolic risk factors, but they are typically brief [13]. Adults gain one-half kilogram per year on average [14], and the effect of avoiding this weight gain over time, especially in high-cardiometabolic risk populations, remains unclear.

Therefore, we performed a systematic review to determine the comparative effectiveness of self-management, diet, and physical activity interventions without a weight loss focus compared with another lifestyle intervention or usual care on weight, cardiometabolic risk factors, and clinical outcomes in adults with or at risk for diabetes or CVD.

## 2. Materials and Methods

The protocol for this study can be found at (http://effectivehealthcare.ahrq.gov/index.cfm/search-for-guides-reviews-and-reports/?productid=824&pageaction=displayproduct) and is based on the Agency for Healthcare Research and Quality (AHRQ) Methods Guide for Effectiveness and Comparative Effectiveness Reviews (http://www.effectivehealthcare.ahrq.gov/methods_guide.cfm). The current study is part of a Comparative Effectiveness Review requested by AHRQ on weight maintenance in adults [15]. This study focuses on weight neutral lifestyle interventions in populations with or at risk for diabetes or CVD.

### 2.1. Eligibility Criteria

We included full text articles with original data that compared the effects of weight neutral (i.e., those not focusing on weight loss as a goal of the study) lifestyle interventions relative to a concurrent comparison group (active intervention listed below, minimal intervention, or usual care) on weight, BMI, or waist circumference in adults with diabetes, CVD, or risk factors for these conditions (e.g., elevated blood sugar, dyslipidemia, and hypertension). Included studies could evaluate interventions for weight maintenance or avoidance of weight gain; if the weight-related focus of a study was unclear, we contacted authors as described below. Active interventions of interest were self-management, diet, physical activity interventions, or combinations of these in which weight loss was not a specified goal. We considered the following to be elements of self-management: problem-solving, addressing barriers, self-monitoring, goal-setting, and individualized counseling. We excluded studies of weight loss; weight maintenance after weight loss; pregnant women; patients at risk for weight loss (e.g., patients on dialysis); smoking cessation; biologic determinants of weight (e.g., genes); supplements; and interventions lasting <12 months. We excluded studies not reporting quantitative results for weight, BMI, or waist circumference. Additional outcomes were adverse events, adherence, and quality of life. Clinical outcomes included mortality, cancer, cardiovascular disease, subfertility, diabetes, degenerative joint disease, liver disease, and quality of life. Although it is not included in the full evidence report [15], for this paper, we also included hemoglobin A1c (HbA1c), blood pressure, low density lipoprotein (LDL) cholesterol, and high density lipoprotein (HDL) cholesterol. We included observational studies that accounted for confounding and losses to follow-up in the weight analyses.

### 2.2. Search

We searched the MEDLINE, Embase, and Cochrane Central Register of Controlled Trials electronic databases through June 2012. We searched the references of included articles and key review articles. We searched ClinicalTrials.gov on June 19, 2012. The MEDLINE search strategy is available in the full report.

### 2.3. Study Selection

Two investigators reviewed titles, abstracts, and full text articles independently. All titles included by one investigator were reviewed at the abstract level. Two investigators agreed on inclusion during the abstract and full text reviews. Disagreements were resolved through consensus. All articles meeting eligibility criteria were included in the systematic review. When an article did not explicitly state a weight-related goal, we contacted the authors for clarification. We excluded studies if the authors responded that weight loss was an intended outcome and included any articles for which we did not receive an author response. We also excluded any articles not reporting on our primary outcomes, weight, BMI, and waist circumference, in their abstracts as we assumed that those weight outcomes were not important measures in those studies.

### 2.4. Data Collection

Data were extracted from articles by two investigators sequentially using forms created in the web-based software, DistillerSR (Evidence Partners, Ottawa, Ontario, Canada). We piloted all forms prior to use. A senior reviewer reviewed the initial abstraction performed by a junior investigator. Disagreements were resolved through consensus. Data on study design, interventions, study population characteristics, outcomes, timing, and setting were abstracted. We collected data on 12-month outcomes and the longest time point available in each study.

### 2.5. Risk of Bias

We evaluated the risk of bias in individual studies using the Downs and Black criteria [31] and focused on reporting and internal validity across studies to assign a rating of “low,” “moderate,” or “high” risk of bias for the weight outcomes. Randomized, controlled trials (RCTs) were considered to be at “low” risk of bias but were downgraded to “moderate” if they did not report masking of outcome assessors for the weight outcomes. Other concerns could also result in downgrading of the risk of bias.

We based our rating of the strength of evidence for each outcome on the risk of bias, consistency (“consistent” if most effect estimates were in the same direction), directness, (“direct” based on explicitly stated a goal of weight gain prevention), and precision (“precise” if statistically significant (*P* < 0.05)) of the studies evaluating that outcome [32]. For adherence, we considered the evidence to be precise if the study included >400 participants. We rated the strength of evidence as “high,” “moderate,” “low,” or “insufficient (no evidence)” based on confidence in the effect estimate as previously described [32].

### 2.6. Synthesis of Results

We did not perform meta-analyses because of the heterogeneity of study interventions. We defined thresholds for clinically meaningful between-group differences over 12 months based on expected increases in anthropometric measures and effects of lifestyle interventions on biochemical measures as follows: weight, 0.5 kg [14]; BMI, 0.2 kg/m^2^ (based on a one-half kg change with initial BMI 27 kg/m^2^); waist circumference, 1 cm [33]; blood pressure, 5 mmHg; HbA1c, 0.7% [34]; HDL cholesterol, 5 mg/dL [35]; and LDL cholesterol, 10 mg/dL [13].

## 3. Results

### 3.1. Search Results

**Figure 1:**
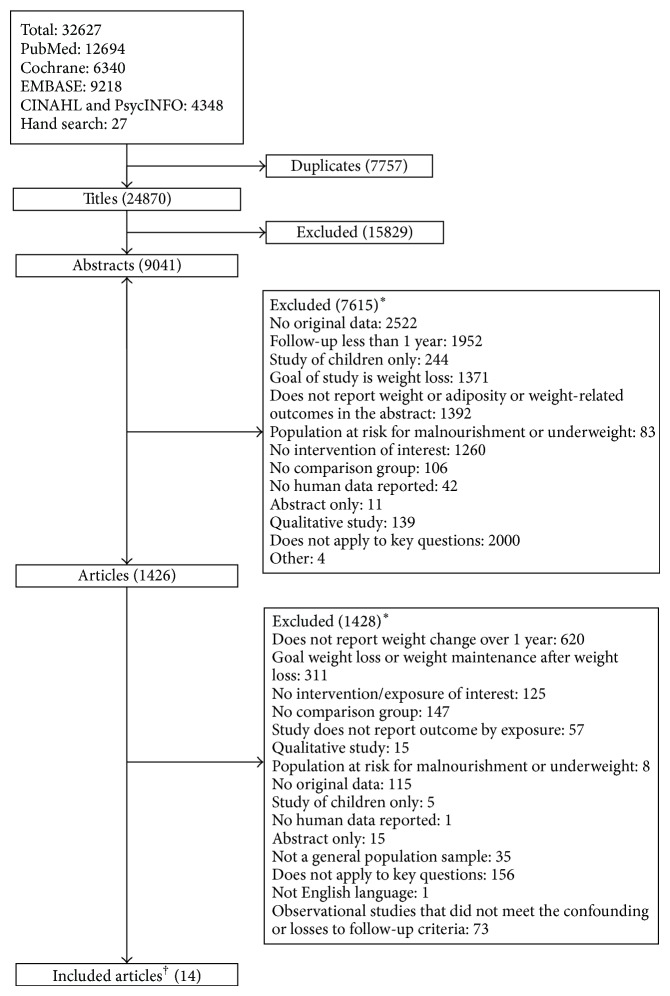
Selection of included studies. ^∗^Sum of reasons for exclusion of abstracts and articles exceeds total number of excluded abstracts and articles because reviewers were not required to agree on reasons for exclusion. ^†^Fourteen articles of 12 individual studies included.

Of 24,870 electronic citations, 9,041 abstracts, and 1,426 full text articles, we identified 11 RCTs [17–29] and one nonrandomized clinical trial [16] evaluating the effect of self-management, dietary, or physical activity interventions without a weight loss focus in persons with or at risk for diabetes and/or CVD (Figure 1). We did not identify observational studies meeting our inclusion criteria.

### 3.2. Study Characteristics

Study characteristics are provided in Table 1. Weight maintenance was a stated goal in one study [16]; the other studies emphasized lifestyle changes and did not explicitly state weight loss as a goal [17–29]. Six studies were conducted in patients with diabetes [16, 20, 21, 23, 26, 28]. The other six studies included participants with risk factors for diabetes or CVD such as dyslipidemia, elevated blood pressure, and elevated HbA1c [17–19, 22, 24, 25, 27, 29]. Four of these excluded patients with CVD [17, 18, 22, 24, 25, 27], and three excluded patients with diabetes [17, 18, 22, 27]. Most trials were conducted in Europe [17, 18, 20, 21, 24, 25, 29] and the United States [16, 22, 28] and at a single study site [16–21, 26, 27, 29]. Most studies did not report on years of recruitment [16, 20, 21, 23–28]. Participants were recruited from a clinical setting in seven studies [16, 20, 21, 23–26, 28] and the others from a cohort study [17, 18], insurance plan [19], and diabetes screening program [29]; two studies did not report on recruitment setting [22, 27]. Follow-up ranged from one to two years [16–18, 23, 26, 28].

The 12 trials enrolled 4,206 participants (see Table S1 in Supplementary Material available online at http://dx.doi.org/10.1155/2014/358919). Women comprised 31 to 100% of participants in the nine studies reporting on sex [19–30]. Mean age ranged from 57 to 68 years in nine trials [16, 19–21, 23–26, 28–30] and was reported by sex in another (57 years for women and 48 years for men) [27]. All participants were Latina in one trial [28]; twenty-six percent Asian or Pacific Islander in another [29]; and 17% black in another [22]. Nine trials did not report on race/ethnicity [16–21, 23–27, 30]. Education level varied across studies with 65% of participants reporting some postsecondary education in one study [23] and less than one-third of participants reporting this in others [24, 25, 28, 30]. One study excluded current smokers [26], and current smoking ranged from 10 to 20% in three studies [24, 25, 28–30].

We identified two trials of self-management interventions [20, 23]; two of dietary interventions [16, 24, 25, 30]; two of physical activity interventions [17, 18, 29]; and six of combinations of these (Table 2) [19, 21, 22, 26, 28].

### 3.3. Risk of Bias

Major issues affecting internal validity were lack of, or lack of reporting on, masking of outcome assessors, prespecified analyses, allocation concealment, and losses to follow-up (Table S2).

We found low strength of evidence for all outcomes for which there was at least one study (Table S3). The risk of bias for each outcome was moderate or high for all outcomes. Frequently, consistency could not be evaluated because there was only a single study. Evidence was indirect because only one study cited weight maintenance as a goal of the study; the other studies did not specify their goal regarding weight, and their intent was clarified by contacting authors as described in the Methods section. Most evidence was imprecise based on small sample size or lack of reporting on variability.

### 3.4. Synthesis of Results

**Figure 2:**
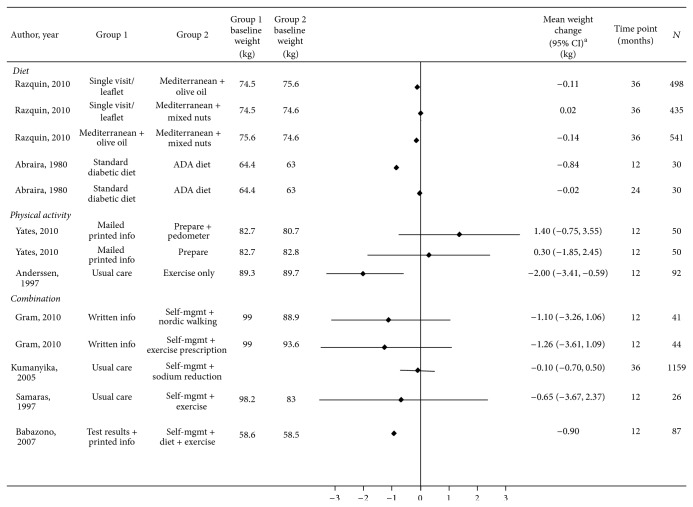
Effect of diet, physical activity, and combination interventions on weight change in participants with or at risk for cardiovascular disease and/or diabetes. ADA: American Diabetes Association; PREPARE: Prediabetes Risk Education and Physical Activity Recommendation and Encouragement. ^∗^CI is not provided if not reported and could not be calculated.

**Figure 3:**
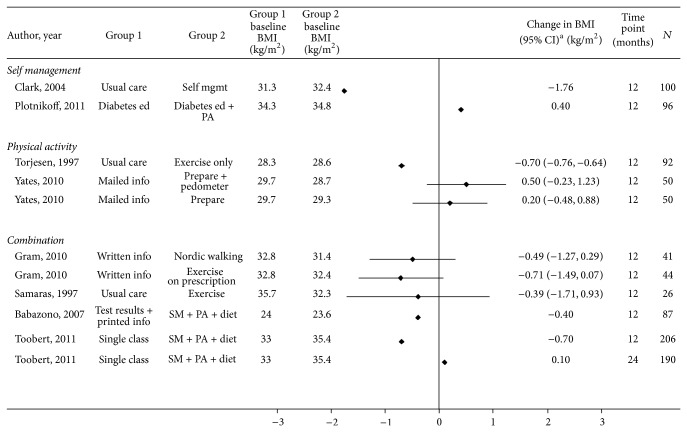
Effect of self-management, physical activity, and combination interventions on BMI change in participants with or at risk for cardiovascular disease and/or diabetes. Mgmt: management; ed: education; PA: physical activity; SM: self-management; PREPARE: Prediabetes Risk Education and Physical Activity Recommendation and Encouragement. ^∗^CI is not provided if not reported and could not be calculated.

**Figure 4:**
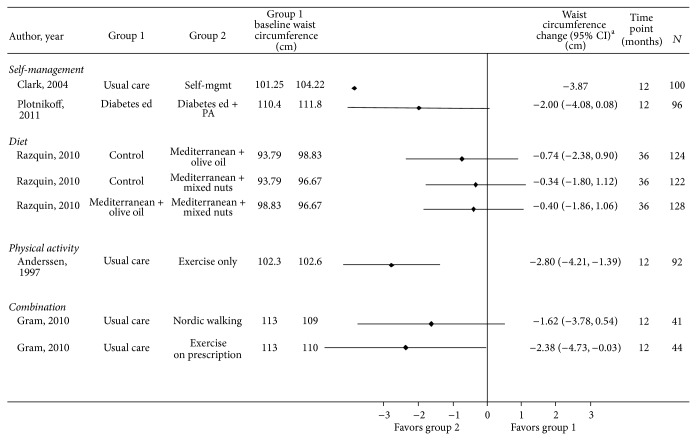
Effect of self-management, diet, physical activity, and combination interventions on waist circumference in participants with or at risk for cardiovascular disease and/or diabetes. Mgmt: management; ed: education; PA: physical activity. ^∗^CI is not provided if not reported and could not be calculated.

*Weight*,* BMI*,* and Waist Circumference*. Study results are provided in Tables 3 and 4 and S4 and Figures 2, 3, and 4.

A self-management intervention based on goal-setting and problem-solving for diet and physical activity prevented an increase in BMI of 1.76 kg/m^2^ at 12 months compared with usual care [20]. A study of an intensive diabetes self-management intervention decreased BMI by 0.4 kg/m^2^ at 12 months compared to the same intervention combined with a physical activity supplement [23]. The statistical significance of findings from these studies was not reported [20, 23]. These studies reported meaningful (between-group differences, −2 to −3.9 cm), but not statistically significant, relative effects of their interventions on waist circumference [20, 23].

In the PREvencion con DIeta MEDiterranea (PREDIMED) Study, two Mediterranean diet interventions did not prevent increases in weight at 36 months [24], BMI at 12 months [30], or waist circumference at 36 months [25] relative to a minimal intervention. The American Diabetes Association diet prevented weight gain relative to a standard diabetic diet at 12 but not 24 months in another study [16, 30].

Walking interventions did not prevent weight or BMI increases at 12 months relative to printed information in the Prediabetes Risk Education and Physical Activity Recommendation and Encouragement (PREPARE) study [29]. In the Oslo Diet and Exercise Study (ODES), relative to control, the endurance exercise intervention prevented meaningful increases (between-group difference (95%CI)) in weight (−2 (−3.4 to −0.6) kg) [17], BMI (−0.7 (−0.76 to −0.64) kg/m^2^) [18], and waist circumference (−2.8 (−4.2 to 1.4) cm) [17] at 12 months.

The combination of self-management with physical activity prevented meaningful increases in weight (−0.65 to −1.26 kg) and BMI (between-group differences range, −0.4 to −0.7 kg/m^2^) versus a comparison group at 12 months in two studies although differences were not statistically significant [21, 26]. The active interventions in one of these studies decreased waist circumference by 1.6 to 2.4 cm at 12 months relative to standard written information (*P* > 0.05) [21].

The combination of self-management, diet, and physical activity led to 0.9 kg more weight loss at 12 months which was not statistically significant compared with a minimal intervention in a single study [19]. Two trials comparing self-management plus exercise and diet decreased BMI relative to a minimal intervention at 12 months (statistical significance not reported) [19, 28], but findings were not sustained at 24 months in the study evaluating this time point [28].

Modest weight gain occurred in both the active intervention arm combining sodium reduction and self-management and in the control arm at 36 months in the Trials of Hypertension (TOHP) II [22].

*HbA1c*. Four studies including patients with diabetes mellitus reported on HbA1c results and did not find significant effects of self-management or combination interventions on HbA1c relative to comparison arms at 12 months (Table S5, Figure S1) [21, 23, 26, 28].

*Blood Pressure*. The combination of self-management and sodium reduction decreased systolic blood pressure by 1.35 mmHg (*P* = 0.0165) and diastolic blood pressure by 0.61 mmHg (*P* = 0.16) relative to control at 36 months in the TOHP II [22]. Studies of self-management alone, physical activity alone, and other combination interventions for weight gain prevention either did not report on [20, 28] or did not significantly affect blood pressure (Table S6, Figures S2-S3) [17, 19, 21, 23]. Studies of diet interventions did not report on blood pressure [16, 30].

*Cholesterol*. Study results are provided in Table S7 and Figures S4-S5. Two active self-management interventions focusing on diabetes management and physical activity each increased HDL cholesterol and decreased LDL cholesterol at 12 months in one study [23]. Exercise interventions did not increase HDL at 12 months relative to comparison groups providing minimal or no intervention in the PREPARE or ODES [17, 29] and did not decrease LDL relative to control in ODES [17]. The combination of self-management and exercise decreased LDL at 12 months in a clinically meaningful but not statistically significant fashion relative to written information in one RCT (between-group difference range, −12 to −16 mg/dL) [21]. In this RCT, the arm incorporating Nordic walking decreased HDL by 6 mg/dL while the arm incorporating an exercise prescription increased HDL by 7 mg/dL relative to the comparison arm; between-group differences were not statistically significant [17]. Studies of dietary interventions did not report on cholesterol [16, 30].

*Adherence*. Adherence to endurance exercise three times per week was 57% over one year in ODES [17]. Adherence to a combination of self-management and physical activity interventions ranged from 64 to 100% [21, 26], and adherence to a combination of self-management, dietary, and physical activity was 46% in one RCT [28]. Trials of self-management and dietary interventions did not report on adherence.

## 4. Discussion

Among adults with or at risk for diabetes and CVD undergoing lifestyle interventions not focused on weight loss, we found that combining self-management and exercise prevented clinically significant increases in weight, BMI, and waist circumference as compared to control; however, none of these results were statistically significant. These interventions differentially included dietary components. We did not find consistent evidence that existing self-management, diet, or physical activity interventions alone prevented increases in weight or BMI when studied relative to usual care or a minimal intervention; however, self-management and physical activity interventions in isolation prevented clinically meaningful, nonsignificant increases in waist circumference. None of the included studies led to a meaningful decrease in HbA1c or blood pressure relative to usual care; however, combining self-management and exercise lead to clinically meaningful, nonsignificant reductions in LDL. Overall, we found moderate-to-high risk of bias and low strength of evidence for weight-related outcomes in this study.

In the United States, adults gain on average one-half kg per year [14], related to a variety of factors that influence energy balance including overconsumption of calories and sedentary behavior, which are reinforced by both environmental and social factors. Many cardiometabolic diseases are negatively influenced by weight gain and obesity; paradoxically, many adults with cardiometabolic disease take prescriptions to manage these conditions that have been associated with weight gain. For example, sulfonylureas, thiazolidinediones, and insulin increase weight [36, 37], and beta-blockers are linked to weight gain [38]. This situation creates a clinical conundrum for providers who counsel their patients to avoid weight gain and yet may prescribe medications which cause just that. Therefore, better understanding what interventions are effective at preventing weight gain and/or maintaining weight among this high risk population is of critical importance.

In counseling patients on preventing weight gain, providers must consider how individual factors such as age, sex, obesity, and type 2 diabetes contribute to variation in the response to exercise and reduced caloric intake. Patients with diabetes undergoing an exercise intervention may not experience the beneficial physiologic changes in adipose tissue such as postexercise mobilization of fatty acids equivalent to that observed in lean, healthy individuals [39], which could explain the general lack of significant differences between active and minimal intervention arms on biochemical measurements such as blood pressure and HbA1c, especially in the absence of significant weight loss. A recent meta-analysis of RCTs in patients with type 2 diabetes did demonstrate the benefit of structured exercise on HbA1c which increased with the number of hours per week spent exercising [40]. The studies included in these meta-analyses were typically less than 12 months in duration and were not restricted to weight neutral interventions [40]. The weight neutral studies of exercise in patients with type 2 diabetes in our review were typically of lower intensity and were >12 months in duration. Similar to type 2 diabetes, obesity also modifies the response of adipose tissue to exercise [39]. Insulin resistance is associated with a hyperadrenergic state [41] which may predispose to resistance to blood pressure reduction with lifestyle interventions. In total, patients with conditions placing them at higher risk for cardiometabolic disease may be in a state of metabolic disarray that attenuates their response to less-intensive physical activity and diet interventions such as those not focused on weight loss.

In this review, physical activity was an essential component of effective interventions with respect to weight gain prevention, weight maintenance, and LDL reduction. In theory, increased energy expenditure through physical activity with or without increases in fat-free mass should result in maintenance of body weight, if not weight loss. Resistance training exercise, as seen in two of the studies reviewed [19, 21], can increase fat-free mass and therefore increase energy expenditure [42]. This increased energy expenditure then facilitates weight gain prevention. Exercise can also reduce fat mass in proportion to the magnitude of the resulting energy deficit, but even substantial increases in physical activity may not cause enough of an energy deficit to result in weight loss [39]. However, exercise facilitates a small energy deficit that can result in weight or fat mass maintenance as observed in several of the included studies. This type of small energy deficit may preferentially reduce visceral fat depots [39, 43], which may lead to improved glycemic and lipid control for these patients independent of weight loss. We would encourage healthcare providers to counsel their patients with or at risk of cardiometabolic disease to regularly engage in both aerobic and resistance training exercise according to the current guidelines [44]. However, future research needs to confirm that the combination of self-management and exercise is the optimal strategy for preventing weight gain among these patients and elucidate specific details such as optimal duration and intensity, types of activities of most benefit, and subgroups (e.g., those with a higher versus lower BMI) which may benefit most.

We found moderate-to-high risk of bias for all main outcomes mainly because of a lack of masking of outcome assessors and other threats to internal validity and a lack of adequate reporting. We identified a total of 12 studies evaluating weight neutral lifestyle interventions in patients with or at risk for CVD and diabetes. Most of these studies were too small to find a statistically significant difference, and heterogeneity of interventions precluded quantitative synthesis of results across studies. Only one [16] of the 12 included studies stated that weight maintenance was the goal of the study. We included all studies meeting our inclusion criteria that did not explicitly state that weight loss was a goal of the study and by being inclusive, we may have included studies that actually targeted weight loss but just did not state this. This inclusion would be expected to bias our results toward showing a benefit of the interventions.

In this systematic review, experienced investigators conducted a thorough search of electronic databases and abstracted and synthesized data using a detailed protocol. Based on the extensive search, it is unlikely that we excluded important studies evaluating our interventions of interest. We are unaware of another systematic review evaluating weight-neutral lifestyle interventions in populations with or at risk for diabetes and/or CVD.

Adults tend to gain weight over time, and this, combined with difficulties translating weight loss interventions into clinical practice, makes the identification of interventions which help individuals avoid weight gain, especially in high-risk populations, paramount. In this systematic review, we found some evidence that the combination of self-management and exercise with or without diet may prevent weight gain or increases in BMI in patients with or at risk for diabetes and/or CVD, although the quality and strength of evidence were low. The downstream effect of these interventions on intermediate and clinical outcomes remains poorly studied. While the overall the evidence is insufficient to determine if weight neutral self-management, physical activity, and dietary interventions are worth pursuing at a population level, the available literature suggests that the combination of self-management and physical activity interventions holds the most promise. Given the lower intensity of interventions targeting avoidance of weight gain and the public health burden of weight gain in adulthood, we recommend future research on weight gain prevention interventions in patients with or at risk for diabetes and CVD to address the limitations of existing evidence and evaluate the impact of weight gain prevention interventions on intermediate and long-term clinical outcomes in these high-risk patients.

## Supplementary Material

The Supplemental Material provides tables with additional details on the included studies and results of our systematic review. Supplemental tables include information on study participant characteristics and individual study results for additional outcomes (waist circumference, hemoglobin A1c, blood pressure, and cholesterol). Also, tables are included which show the risk of bias (quality) assessments for each included study and details on the Strength of Evidence grading for the body of literature included in this systematic review.

## Figures and Tables

**Table 1:** Characteristics of studies on populations with or at risk for diabetes mellitus and cardiovascular disease.

Author, year Study location	Years of recruitment	Single or multicenter	Recruitment setting	Study design	Inclusion criteria			
					Age	Body mass index	Diabetes mellitus	Other
Abraira et al., 1980 [16] United States	Not reported	Single	Clinical	Nonrandomized			DM on insulin	Veteran

Anderssen et al., 1995 [17] Torjesen et al., 1997 [18] Europe	1990-1991	Single	Cohort study in Oslo	Randomized	41–50 years	>24 kg/m^2^		Relatively inactive DBP of 86–99 mmHg

Babazono et al., 2007 [19] Asia	2004	Single	Membership	Randomized				SBP 130–159 mmHg, DBP 85–99 mmHg, or hemoglobin A1c >5.6%

Clark et al., 2004 [20] Europe	Not reported	Single	Clinical	Randomized	40–70 years	>25 kg/m^2^	Type 2 DM	Safe to participate in walking program

Gram et al., 2010 [21] Europe	Not reported	Single	Newspaper and clinical	Randomized	25–80 years	>25 kg/m^2^	Type 2 DM for >1 year	Hemoglobin A1c 7% to 10%

Kumanyika et al., 2005 [22] United States	1992	Multicenter	Not reported	Randomized	30–54 years			No evidence of CVD, DM, renal insufficiency, or other serious illness

Plotnikoff et al., 2011 [23] Canada	Not reported	Not reported	Diabetes education programs	Randomized			Type 2 DM	

Razquin et al., 2010 [24] Razquin et al., 2009 [25] Europe	Not reported	Multicenter	Clinical	Randomized	Men 55–80, women 60–80 years	>25 kg/m^2^	Type 2 DM	No history of CVD No intolerance to olive oil or nuts

Samaras et al., 1997 [26] Australia	Not reported	Single	Clinical	Randomized	40–70 years		Diabetes not requiring insulin	Perform <1 hour exercise weekly No history/symptoms or signs of IHD no smoking

Stefanick et al., 1998 [27] Location not reported	Not reported	Single	Not reported	Randomized	Women 45–64, men 30–64 years	Men < 34, women < 32 kg/m^2^	No DM	No history of disease limiting their ability to engage in moderate-intensity exercise

Toobert et al., 2011 [28]	Not reported	Multicenter	Clinical	Randomized	30–75 years		Type 2 DM	English or Spanish speaking Latino/Hispanic ethnicity

Yates et al., 2010 [29] Europe	2006-2007	Single	Diabetes screening programs	Randomized		>25 kg/m^2^ (>23 kg/m^2^ for South Asians)	Impaired glucose tolerance	Not taking steroids

DM: diabetes mellitus; DBP: diastolic blood pressure; SBP: systolic blood pressure; CVD: cardiovascular disease; IHD: ischemic heart disease.

**Table 2:** Description of interventions to prevent weight gain in populations with or at risk for diabetes and cardiovascular disease.

Author, year Duration	Primary aim	Control group	Active Intervention 1	Active Intervention 2
**Self-management **				

Clark et al., 2004 [20] 6 months	Goal-setting for diet and physical activity using motivational interviewing	Usual care/no intervention	(i) 30 min in-person session at baseline to set goals/address barriers (ii) 10 min in-person session at 3 and 6 mo to problem-solve and set additional goals (iii) Telephone contacts at 1, 3, and 7 wks	(i) Not applicable

Plotnikoff et al., 2011 [23] 12 months	Goal-setting for physical activity and/or diet using social-cognitive teaching	Not applicable	(i) Standard Diabetes Education Program (DEP) from local health authority (ii) Goal: increase physical activity to meet Canadian Diabetes Association recommendations (iii) Social-cognitive approach (iv) 8 group sessions over 4 wks (12 hrs) on self-care (v) Follow-up group sessions at 3, 6, and 12 mo (vi) Telephone support by diabetes educator	(i) DEP + 8-week physical activity supplement (ii) Certified personal trainer (a) Individualized counseling and prescription tailored to fitness level and stage of change and grounded in Social Cognitive Theory (iii) Free 2 mo membership to a community recreational facility or “at-home” program (a) Personal trainer facilitated (iv) Telephone support by trainer

**Dietary **				

Zazpe et al., 2008 [30] Razquin et al., 2010 [24] Razquin et al., 2009 [25] 36 months	Mediterranean diet	Leaflet + single meeting with dietician on American Heart Association dietary recommendations	(i) Mediterranean diet with emphasis on virgin olive oil (ii) Quarterly individual sessions with dietician with motivational interviewing (iii) Group educational sessions (iv) Free access to study center dietician (v) Free olive oil	(i) Same as Active Intervention 1 but with free provision of mixed nuts instead of olive oil

Abraira et al., 1980 [16] 24 months	Change diet	Not applicable	Standard diabetic diet (i) Three meals + bedtime snack (ii) Strict avoidance of refined sugars (iii) Allowed consumption of starches (iv) Avoidance of saturated fat (v) No exchange system (vi) No caloric goal (vii) No specific carbohydrate distribution (viii) Quarterly visits with dietician	American Diabetes Association diet (i) Three meals + bedtime snack (ii) Moderate restriction of refined sugars and carbohydrates (iii) Daily meal pattern planned and distributed through a food exchange (iv) Daily caloric goal (v) Specific carbohydrate distribution (vi) Quarterly visits with dietician

**Physical activity **				

Yates et al., 2010 [29] 6 months	Increase physical activity through walking	Mailing on impaired glucose tolerance and physical activity	(i) 180 min group session at baseline-information on impaired glucose tolerance; counseling on exercise, self-efficacy beliefs, barriers to walking, and self-regulatory strategies (ii) 10 min review of progress in-person at 3 and 6 mo (iii) Steps per day goal and pedometer	(i) Same as Active Intervention 1 but no pedometer given

Anderssen et al., 1995 [17] Torjesen et al., 1997 [18] 12 months	Increase peak VO_2 _through endurance exercise	Usual care/no intervention	(i) Supervised exercise sessions: 60 min three times per week (ii) Goal: improve peak VO_2_-target 60–80% of peak heart rate	(i) Not applicable

**Combination **				

Samaras et al., 1997 [26] 6 months	Increase physical activity	Usual care/no intervention	Self-management (i) Monthly in-person session: education; coping skills; improving confidence, self-esteem, decision-making, and goal-setting (a) Hand-outs, videos, activity meters, log books for goal-setting, and review of progress Physical Activity (i) Monthly in-person aerobic exercise session with exercise physiologist (ii) Goal: 50 percent of peak VO_2_ by perceived exertion (iii) Exercise sessions after 6 mo intervention period	(i) Not applicable

Gram et al., 2010 [21] 4 months	Increase physical activity	Written advice on exercise	Self-management (i) In-person interviews at 0, 8, 16, and 24 wks for goal-setting and tailored advice Physical activity (i) In-person supervised exercise sessions (45 min) (ii) Focus on strength training and aerobic exercise (iii) Access to exercise equipment (iv) Goal: >40% of peak VO_2_ by perceived exertion (v) Encouraged activity outside of training sessions (vi) Information on physical training in neighborhood at end of intervention period	Self-management component (i) Same as Active Intervention 1 Physical activity component (i) Same as Active Intervention 1 except Nordic walking (ii) Received walking sticks with individualized stick length

Babazono et al., 2007 [19] 12 months	Increase fruits, vegetables, and physical activity	Received result of health exam; leaflet about exercise; and having 3 conventional health center visits without additional services	Self-management (i) Received results of health exam (ii) 5 in-person sessions at health center to set personal diet and physical activity goals, problem solve, and receive advice (iii) 3 health center visits + 2 home visits Diet (i) Increase fruits/vegetables; decrease salt, oil, sugar, and alcohol; and increase time for meals, eat more slowly Physical activity (i) Challenge cards to increase activity	(i) Not applicable

Stefanick et al., 1998 [27] 9–11 months	Follow NCEP diet and/or increase aerobic exercise^*^	Usual care/no intervention: asked to maintain usual diet and exercise	Diet (i) Follow NCEP step 2 diet (ii) 12-week adoption phase: one counseling session and 8 one-hour group lessons (iii) Maintenance: monthly contact with dietician by mail, telephone, or in-person individual or group meetings	Physical activity (i) Aerobic exercise (ii) 6-week adoption phase: single private meeting with exercise staff; in-person, supervised, one-hour exercise session 3 times per week (iii) Maintenance phase: 10 miles of walking/jogging each week; monthly group session; optional continued supervised exercise sessions; optional home activities

Kumanyika et al., 2005 [22] 36–48 months	Consume <1800 mg of sodium/day	Usual care/no intervention	Self-management (i) Intensive phase: initial individual counseling session and 10 weekly group sessions (ii) Transitional phase (a) 4 monthly group sessions + as needed (b) Individual in-person, telephone, and mail contacts as needed (c) Relapse prevention techniques; feedback on urine sodium; self-monitoring; counselor and peer support Diet (i) Consume <1800 mg Na+/day (ii) No change in other dietary intake	(i) Not applicable

Toobert, 2011 [28] 24 months	Mediterranean diet; exercise; smoking cessation; stress management	Usual diabetes care + one free Kaiser-Permanente class targeting goals of the active intervention	Culturally adapted for Latinas (i) 2.5-day retreat (a) Catered Mediterranean meals; physical activity; stress management; support groups; smoking cessation (ii) In-person meetings (a) Weekly for 6 mo, biweekly for mo 6–12, monthly for mo 12–18, and every other mo for mo 18–24 (b) Mediterranean meal potluck; physical activity; stress management; support groups; family nights Self-management (i) Stress-management techniques for at least 60 minutes/day (a) Group support for 60 minutes/each meeting (b) Mini-units on goal-setting, social support, problem solving, negative thoughts, and barriers (ii) Smoking cessation Mediterranean diet (i) Catered events, potlucks, and demonstrations Exercise (i) 30 min moderate aerobic activity most days (ii) 10 strength-training exercises two times per wk	(i) Not applicable

Min: minutes; mo: month; hr: hour; wk: week; NCEP: National Cholesterol Education Program.

^*^Active Intervention 3: Active Intervention 1 + Active Intervention 2 (Diet + Physical Activity).

**Table 3:** Weight results in studies of populations with or at risk for diabetes mellitus and cardiovascular disease.

Author, year Intervention	Baseline *N*	Baseline weight, mean (SD), kg	*N* at 12 months	Weight, 12 months, mean (SD), kg	Change from baseline (95% CI)	Between-group difference in change from baseline at 12 months (95% CI), kg
Diet						
Razquin et al., 2010 [24]^*^						
Minimal intervention	196	74.5 (11.8)			−0.10 (SE: 0.3)	Reference
Mediterranean-virgin olive oil	302	75.6 (11.9)			−0.21 (SE: 0.2)	−0.11
Mediterranean-mixed nuts	239	74.6 (10.3)			−0.07 (SE: 3.8)	0.02
Abraira et al., 1980 [16]						
Standard diabetic diet		63.0			1.58	Reference
ADA diet		64.4			0.74	−0.8
Physical activity						
Yates et al., 2010 [29]						
Minimal intervention	26	82.7 (14.7)	26	81.9	−0.8 (−2.3 to 0.6)	Reference
Walking + pedometer	24	80.7 (17.2)	24	81.2	0.5 (−1.2 to 2.2)	1.4 (−0.8 to 3.5); *P* = 0.199^†^
Walking	24	82.8 (14.6)	24	82.3	−0.5 (−2.1 to 1.1)	0.3 (−1.85 to 2.45)^†^
Anderssen et al., 1995 [17]						
Usual care	43	89.3 (SE: 2.1)	43	90.4	1.1 (SE: 0.4)	Reference
Endurance exercise	49	89.7 (SE: 1.7)	49	88.8	−0.9 (SE: 0.7)	−2 (−3.4 to −0.6); *P* = 0.007
Combination						
Kumanyika et al., 2005 [22]						
Usual care^‡^	577					
Self-mgmt + sodium reduction^§^	582					
Samaras et al., 1997 [26]						
Usual care	13	98.2 (SE: 3.4)			0.79 (SE: 1.09)	Reference
Self-mgmt + aerobic exercise	13	83.0 (SE: 3.6)			0.14 (SE: 1.09)	−0.65 (−3.67 to 2.37)
Babazono et al., 2007 [19]						
Minimal intervention	41	58.6 (9.1)	41	58.1 (8.8)	−0.5	Reference
Self-mgmt + diet + physical activity	46	58.5 (9.7)	46	57.1 (9.5)	−1.4	−0.9
Gram et al., 2010 [21]						
Minimal intervention	22	99 (15)	20	98.8 (SE: 3.2)		Reference
Self-mgmt + aerobic exercise + strength training	24	93.6 (14.8)	24	92.5 (SE: 3.2)		−1.26 (−3.61 to 1.09)
Self-mgmt + Nordic walking	22	88.9 (14.3)	21	87.1 (SE: 3.3)		−1.1 (−3.26 to 1.06)
Stefanick et al., 1998 [27]						
All women		69.6 (10.5)				
Usual care	45		45		0.8 (4.2)^‡‡^	Reference
Aerobic exercise	43		43		−0.4 (2.5)	−1.2
NCEP step 2 diet	46		46		−2.7 (3.5)	−3.5; *P* < 0.001^**^
NCEP step 2 diet + aerobic exercise	43		43		−3.1 (3.7)	−3.9; *P* < 0.001^††^
Stefanick et al., 1998 [27]						
All men		84.2 (10.8)				
Usual care	46		46		0.5 (2.7)^‡‡^	Reference
Aerobic exercise	47		47		−0.6 (3.1)	−1.4
NCEP step 2 diet	49		49		−2.8 (3.5)	−3.6; *P* < 0.001^§§^
NCEP step 2 diet + aerobic exercise	48		48		−4.2 (4.2)	−5.0; *P* < 0.001^***^

SD: standard deviation; SE: standard error; mgmt.: management; NCEP: National Cholesterol Education Program.

^*^36 mo body weight change (36 mo versus baseline for Arm 2 versus Arm 1): 0.20 kg (95% CI: −0.593 to 0.997 kg); *P* = 0.618.

^†^Adjusted for weight.

^‡^Mean change from baseline at 36 mo (SD): 1.8 (5.3) kg.

^§^Mean change from baseline at 36 mo (SD): 1.7 (2); mean between-group change at 36 mo: −0.1, SE: 0.31, *P* = 0.75.

^**^ *P* < 0.05 versus aerobic exercise arm.

^††^ *P* < 0.01 versus aerobic exercise arm.

^‡‡^ *P* < 0.001 from ANOVA comparing weight change across all 4 arms; *P* values Bonferroni-adjusted.

^§§^ *P* < 0.05 versus aerobic exercise arm.

^***^ *P* < 0.001 versus aerobic exercise arm.

**Table 4:** BMI results in studies of populations with or at risk for diabetes mellitus and cardiovascular disease.

Author, year	Baseline *N*	Baseline BMI, mean (SD), kg/m^2^	*N* at 12 months	12 months, mean (SD), kg/m^2^	BMI change at 12 months, mean (SD), kg/m^2^	Between-group difference in change from baseline at 12 months (95% CI), kg/m^2^
Self-management						
Clark et al., 2004 [20]						
Usual care	50	31.3 (5.01)	50	32.72 (4.77)	1.42	Reference
Goal-setting diet and physical activity	50	32.4 (4.49)	50	32.06 (4.03)	−0.34	−1.76
Plotnikoff et al., 2011 [23]						
Diabetes education	49	34.8 (9)	49		−1.2	Reference
Diabetes education + physical activity supplement	47	34.3 (5.7)	47		−0.8	0.40
Diet						
Zazpe et al., 2008 [30]						
Minimal intervention^*^	485	29.5 (SE: 3.6)	485			
Mediterranean diet-virgin olive oil^†^	533	29.3 (SE: 3.5)	533			
Mediterranean diet-mixed nuts^‡^	533	29.4 (SE: 3.4)	533			
Physical activity						
Torjesen et al., 1997 [18]						
Usual care	43	28.3 (3.1)	43		0.4 (0.1)	Reference
Endurance exercise	49	28.6 (3.1)	49		−0.3 (0.2)	−0.7 (−0.8 to −0.6); *P* < 0.001
Yates et al., 2010 [29]						
Minimal intervention	26	29.7 (4.5)			−0.3 (95% CI: −0.8 to 0.2)	Reference
Walking + pedometer	24	28.7 (5)			0.1 (95% CI: −0.5 to 0.7)	0.5 (−0.3 to 1.2); *P* = 0.21^§^
Walking	24	29.3 (5.1)			−0.1 (95% CI: 0.6 to 0.4)^**^	0.2 (−0.5 to 0.9); *P* = 0.58^§^
Combination interventions						
Gram et al., 2010 [21]						
Minimal intervention	22	32.8 (4.0)	20	32.6 (SE: 0.9)		Reference
Self-mgmt + aerobic exercise + strength training	24	32.4 (4.1)	24	31.8 (SE: 0.9)		−0.71 (−1.49 to 0.07); *P* = 0.049
Self-mgmt + Nordic walking	22	31.4 (4.3)	21	30.9 (SE: 0.9)		−0.49 (−1.27 to 0.29)
Samaras et al., 1997 [26]						
Usual care	13	35.7 (SE: 1.6)			0.29 (SEM: 0.45)	Reference
Self-management + physical activity	13	32.3 (SE: 1.1)			−0.1 (SEM: 0.05)^††^	−0.39 (−1.71 to 0.93)
Babazono et al., 2007 [19]						
Minimal intervention	41	24 (2.5)	41	23.9 (2.4)	−0.1	Reference
Self-management + diet + physical activity	46	23.6 (3.2)	46	23.1 (3.2)	−0.5	−0.4

BMI: body mass index; SD: standard deviation; SE: standard error; mgmt.: management.

^*^% with reduction in BMI = 41.2.

^†^% with reduction in BMI = 37.7.

^‡^% with reduction in BMI = 40.9.

^§^Adjusted for BMI.

^**^Lower limit for confidence interval for Arm 3 should probably be −0.6 rather than +0.6 (likely typographical error).

^††^No significant difference by arm with ANOVA or Mann-Whitney test.
