# Supplementary material for: Avoiding Weight Gain in Cardiometabolic Disease: A Systematic Review
Source: J Obes. 2014 Dec 28;2014:358919. doi: 10.1155/2014/358919 (PMC4291140; doi:10.1155/2014/358919)
Supplement: Supplementary file 1 — The Supplemental Material provides tables with additional details on the included studies and results of our systematic review. Supplemental tables include information on study participant characteristics and individual study results for additional outcomes (waist circumference, hemoglobin A1c, blood pressure, and cholesterol). Also, tables are included which show the risk of bias (quality) assessments for each included study and details on the Strength of Evidence grading for the body of literature included in this systematic review. [file 358919.f1.pdf]

SUPPLEMENTAL MATERIAL

Avoiding Weight Gain in Cardiometabolic Disease: A Systematic Review

Nisa M Maruthur, MD, MHS[1,2,3]
Kimberly Gudzune, MD, MPH[1,2]
Susan Hutfless, PhD[4]
Oluwakemi A Fawole, MBCHB, MPH[1]
Renee F Wilson, MS[5]
Brandyn D Lau, MPH, CPH[6]
Cheryl A M Anderson, PhD[2,3,7]
Sara N Bleich, PhD[5]
Jodi Segal, MD, MPH[1,5]

Corresponding author: Nisa M. Maruthur, MD, MHS; 2024 E. Monument St., Suite 2-600, Baltimore, MD, 21287; telephone 410-502-8896, fax 410-955-0476; email: maruthur@jhmi.edu

[1] Division of General Internal Medicine, Johns Hopkins University School of Medicine, Baltimore, Maryland
[2] Welch Center for Prevention, Epidemiology, and Clinical Research, Baltimore, Maryland
[3] Department of Epidemiology, Johns Hopkins University School of Medicine, Baltimore, Maryland
[4] Division of Gastroenterology, Johns Hopkins University School of Medicine, Baltimore, Maryland
[5] Department of Health Policy and Management, Johns Hopkins Bloomberg School of Public Health, Baltimore, Maryland
[6] Division of Acute Surgery, Department of Surgery, Johns Hopkins University School of Medicine, Baltimore, Maryland
[7] Department of Family and Preventive Medicine, University of California at San Diego, San Diego, California

Supporting Tables

Table S1. Participant characteristics in studies of patients with or at risk for diabetes and cardiovascular disease

Author, year	Total N	Follow-up period	% Women	Mean Age, yrs	Race	Education	% Current Smoking	Comments
Abraira, 1980[1]	30	24 months	0	Arm 1: 49<br>Arm 2: 52				Duration of diabetes in years<br>Arm1: mean : 11<br>Arm2: mean : 12<br>All participants on insulin
Anderssen, 1995[2]<br>Torjesen, 1997[3]	97	12 months						
Babazono, 2007[4]	99	Not reported	Arm1: 51.1<br>Arm2: 58	Arm1: 64.5<br>Arm2: 64.3				~30% with HTN at baseline in each group; 20% in intervention and 14.3% in control group with DM at baseline
Clark, 2004[5]	100	Not reported	Overall: 42	Overall: 59.5				
Gram, 2010[6]	68	Not reported	Overall: n=31	Arm1: 61<br>Arm2: 59<br>Arm3: 62				At baseline: statistically significant difference (P=0.04) for hip circumference (in cm):<br><br>Arm 1 Control: 114<br>Arm 2 EP: 111<br>Arm 3 NW: 107
Kumanyika, 2005[7]	1159	Not reported	"approx two thirds were male"		Black: 17%	"about half" college graduates		
Plotnikoff, 2011[8]	96	12 Months	Overall: 60	Overall: 60 (25-78)	NR	Some post secondary: 65%	NR	
Razquin, 2009[9]	187	Not reported	Arm1: 54	Arm1: 69				

			Arm2: 52<br>Arm3: 46	Arm2: 67.48<br>Arm3: 68.40				
Razquin, 2010[10]	737	Not reported	Arm1: 57<br>Arm2: 56<br>Arm3: 52	Arm1: 68.3<br>Arm2: 67.7<br>Arm3: 67.6			Arm1: 18<br>Arm2: 16<br>Arm3: 13	Diabetes<br>Arm 1: 69%<br>Arm 2: 64%<br>Arm 3: 63%
Zazpe, 2008[11]	1776	Not reported	Arm1: 57.7<br>Arm2: 53.8<br>Arm3: 49	Arm1: 68<br>Arm2: 67.2<br>Arm3: 67	Not reported	Less than high school<br>Arm1: 75.9%<br>Arm2: 76.0%<br>Arm3: 72.6%	Arm1: 16.9<br>Arm2: 19.5<br>Arm3: 19.0	Diabetes<br>Arm1: 47.8%<br>Arm2: 51%<br>Arm3: 47.5%
Samaras, 1997[12]	26	12 months	Overall: n=16	Arm1: 60.5<br>Arm2: 60.5				Excluded current smokers
Stefanick, 1998[13]	367	Not reported	report characteristics stratified by sex	Arm1: 56.9<br>Arm2: 47.8<br><br>note this is stratified by sex and not intervention				All characteristics and results stratified by sex
Toobert, 2011[14]	280	6-12 months	Overall: 100%	Arm 1: 58.7<br>Arm 2: 55.6<br><br>Age differs by group<br>P=0.009	Latina: 100%	HS diploma:<br>Arm 1: 27.1<br>Arm 2: 20<br><br>Some College:<br>Arm 1: 30.1<br>Arm 2: 28.6	Arm 1: 12.6<br>Arm 2: 9.2	
Yates, 2010[15]	74	Not reported	Overall: 31	Overall: 65	Asian/Pacific Islander overall: % : 26		Overall: 11	49% were taking aspirin, 7% were taking non-steroidal anti-inflammatory medication, 58% were taking statins, 34% were taking beta-blockers, and 19% were taking angiotensin-converting enzyme inhibitors.

Table S2. Risk of bias in included studies

	QUESTION*																											
	**Reporting**											**External validity**			**Internal Validity-bias**							**Internal Validity-confounding and selection bias**						**Power**
	**N**	**1**	**2**	**3**	**4**	**5**	**6**	**7**	**8**	**9**	**10**	**11**	**12**	**13**	**14**	**15**	**16**	**17**	**18**	**19**	**20**	**21**	**22**	**23**	**24**	**25**	**26**	**27**
**Self Management**																												
Clark, 2004[5]	100	Y	N	N	N	Y	N	N	N	Y	Y	U	U	N	U	U	N	Y	Y	Y	Y	Y	Y	U	N	Y	N	Y
Plotnikoff, 2011[8]	96	Y	Y	Y	Y	Y	Y	N	N	N	U	U	Y	U	U	U	U	Y	U	Y	Y	Y	Y	U	Y	N	Y	Y
**Diet**																												
Abraira, 1980 [1]	30	Y	Y	Y	Y	P	N	Y	Y	N	N	U	U	Y	N	N	U	N	Y	Y	Y	Y	Y	N	N	N	U	N
Razquin, 2010 [10]	737	Y	Y	Y	Y	Y	Y	Y	Y	N	Y	U	U	U	U	U	U	N	Y	Y	Y	Y	U	Y	Y	Y	N	N
Razquin, 2009 [9]	187	Y	Y	Y	Y	P	Y	Y	Y	N	N	U	U	U	N	U	U	N	Y	N	Y	Y	U	Y	Y	N	N	N
**Exercise**																												
Yates, 2010 [15]	74	Y	Y	Y	Y	P	Y	Y	N	Y	Y	U	U	U	U	U	Y	Y	Y	U	Y	Y	Y	Y	U	N	N	N
Torjesen, 1997 [3]	219	Y	Y	N	Y	P	Y	Y	N	Y	N	U	U	N	N	N	U	N	Y	Y	Y	Y	Y	Y	U	N	N	N
**Combination**																												
Kumanyika, 2005 [7]	1159	Y	Y	Y	Y	Y	Y	Y	N	N	N	U	U	U	N	Y	N	N	Y	Y	Y	Y	Y	Y	Y	Y	U	Y
Samaras, 1997 [12]	26	Y	Y	Y	Y	Y	N	Y	N	N	N	U	U	Y	U	U	Y	N	Y	Y	Y	Y	U	Y	U	N	U	N
Stefanick, 1998 [13]	367	Y	Y	Y	Y	P	Y	Y	N	Y	Y	U	U	U	N	U	Y	U	Y	U	Y	U	U	Y	U	U	Y	N
Babazono, 2007 [4]	99	Y	Y	Y	Y	P	Y	Y	N	N	N	Y	U	Y	N	U	U	N	Y	N	Y	Y	Y	Y	U	N	N	N
Toobert, 2011 [14]	280	Y	Y	Y	Y	Y	Y	N	Y	Y	U	N	Y	N	U	Y	U	Y	N	Y	Y	Y	Y	U	Y	Y	N	Y
Gram, 2010 [16]	68	Y	Y	Y	Y	Y	Y	Y	Y	N	N	U	U	U	U	U	U	N	Y	Y	Y	Y	U	Y	U	Y	Y	Y

Table S3. Strength of evidence of studies among adults with or at risk for cardiovascular disease or diabetes mellitus

Number of Studies, Participants	Domains Pertaining to Strength of Evidence				Strength of evidence<br><br>Range of between-group mean difference if available (reference = control)
BMI change					
Self-Management interventions	Risk of Bias	Consistency	Directness	Precision	
2 RCTs<br><br>196	Moderate based on lack of reporting on masking of outcome assessors and lack of adequate reporting	Consistent	Indirect (weight maintenance not stated goal)	Imprecise based on lack of reporting on variability	Low<br><br>Range: -1.76 kg/m^2
Diet interventions	Risk of Bias	Consistency	Directness	Precision	
1 RCT<br><br>1551	Moderate based on lack of reporting on masking and lack of internal validity based on q14-27	Not applicable (one study)	Indirect (weight maintenance not stated goal)	Imprecise (no measure of variability)	Low<br><br>Range: not available
Physical activity interventions	Risk of Bias	Consistency	Directness	Precision	
2 controlled trials<br><br>166	Moderate based on lack of reporting on masking of outcome assessors and completers analysis for 1	Inconsistent (based on different signs for between group differences)	Indirect	Imprecise	Low<br><br>Range: -0.2 to -0.7 kg/m^2

	study				
Combination interventions	Risk of Bias	Consistency	Directness	Precision	
4 RCTs<br><br>384	Moderate based on lack of reporting on masking and lack of internal validity based on q14-27	Consistent	Indirect (weight maintenance not stated goal)	Imprecise based on lack of reporting on variability and width of CI > 0.8 units when provided	Low<br><br>Range: -0.39 to -0.71 kg/m^2
Weight change					
Diet intervention	Risk of Bias	Consistency	Directness	Precision	
2 controlled trials<br><br>767	High based on lack of randomization, lack of reporting on masking, and lack of internal validity by q14-27	Consistent	Indirect (weight maintenance not stated goal in one study but was in the other)	Imprecise (don't have enough measures of variability)	Low<br><br>Range: -0.11 to -0.84 kg
Physical activity intervention	Risk of Bias	Consistency	Directness	Precision	
2 controlled trials<br><br>166	Moderate based on lack of reporting on masking of outcome assessors and completers analysis for 1 study	Inconsistent	Indirect	Imprecise (on cusp with one study being imprecise and one not based on 2.5 kg width of CI)	Low<br><br>Range: -0.3 to -2.0 kg
Combination intervention	Risk of Bias	Consistency	Directness	Precision	
4 controlled trials	Moderate based on lack	Consistent	Indirect	Imprecise	Low

1719	of masking				Range: -0.1 to -1.26 kg
Waist circumference					
Self-Management interventions	Risk of Bias	Consistency	Directness	Precision	
2 RCTs<br><br>196	Moderate based on lack of reporting on masking of outcome assessors and lack of adequate reporting	Consistent	Indirect (weight maintenance not stated goal)	Imprecise based on lack of reporting on variability and width of CI > 2 cm when provided	Low<br><br>Range: -3.87 cm
Diet intervention	Risk of Bias	Consistency	Directness	Precision	
1 controlled trial<br><br>187	Moderate based on lack of reporting on masking of outcome assessors and lack of adequate reporting	Not applicable (one study)	Indirect (weight maintenance not stated goal)	Imprecise based on CI >2cm	Low<br><br>Range: -0.34 to -0.74 cm
Physical activity intervention	Risk of Bias	Consistency	Directness	Precision	
1 controlled trial<br><br>92	Moderate risk of bias based on lack of masking of outcome assessors	Not applicable (one study)	Indirect	Imprecise	Low<br><br>Range: -2.8 cm
Combination intervention	Risk of Bias	Consistency	Directness	Precision	
1 RCT<br><br>68	Moderate risk of bias based on lack of reporting on masking of	Not applicable (one study)	Indirect	Imprecise	Low<br><br>Range: -2.38 cm

	outcome assessors				
Adherence					
Physical activity intervention	Risk of Bias	Consistency	Directness	Precision	
1 controlled trial<br><br>92	Moderate risk of bias based on lack of masking of outcome assessors	Not applicable (one study)	Indirect	Imprecise (N<400)	Low<br><br>Range: 57%
Combination					
3 RCT<br><br>191	Moderate based on lack of reporting on masking and lack of internal validity based on q14-27	Inconsistent (by magnitude since is adherence, sign is not applicable)	Indirect (weight maintenance not stated goal)	Imprecise (N<400)	Low<br><br>Range: 46% to 100%
Functional Status					
Combination intervention	Risk of Bias	Consistency	Directness	Precision	
1 controlled trial<br><br>68	Moderate risk of bias based on lack of masking of outcome assessors	Not applicable (one study)	Indirect	Imprecise	Low<br><br>Range: Not available

Table S4. Waist circumference results in studies of populations with or at risk for diabetes and cardiovascular disease

Author, yr<br>Arm	Baseline N	Baseline waist circumference (SD), cm	12 mo N	Waist circumference at 12 months (SD), cm	Change from baseline at 12 mo, cm	Between-group difference at 12 months (95% CI), cm
**Self-management intervention**						
**Clark, 2004** [5]						
Usual care	50	101.25 (11.4)	NR	103.6 (10.63)	2.35, P=0.001	reference
Goal-setting - diet and physical activity	50	104.22 (10.46)	NR	102.7 (10.25)	-1.52, P=0.002	-3.87 (-2.1 to 9.9)
**Plotnikoff, 2011** [8]						
Diabetes education	49	110.4 (12.7)	49		-3.2[a], P<0.01	P>0.01
Diabetes education + physical activity supplement	47	111.8 (16.7)	47		-5.2[a], P<0.01	
**Physical activity intervention** [17]						
**Anderssen, 1995** [2]						
Usual care	43	102.3 (SE: 1.4)	43	103.2	0.9 (SE: 0.4)	
Endurance exercise	52	102.6 (SE: 1.4)	52	100.7	-1.9 (SE: 0.6)	-2.8, P<0.05
**Diet interventions**						
**Razquin, 2009** [9b]						
Minimal intervention	59	93.79 (9.78)				
Mediterranean diet - Virgin olive oil	65	98.83 (10.14)				
Mediterranean diet - Mixed nuts	63	96.67 (9.30)				
**Combination interventions**						
**Gram, 2010** [6]						
Minimal intervention	22	113 (10)	20	112 (SE: 2)		
Self-mgmt + aerobic exercise + strength training	24	110 (10)	24	108 (SE: 2)	-2.38 (95% CI: -4.73 to -0.03; SE: 1.2; P=0.012)	
Self-mgmt + Nordic walking	22	109 (11)	21	108 (SE: 2)	-1.62 (95% CI: -3.76 to 0.52; SE: 1.1)	

[a] Adjusted but covariates unclear

[b] Results at 36 months: Minimal intervention, n=59, mean change from baseline (SD), 0.11 (4.56) cm; Mediterranean - virgin olive oil, n=65, mean change from baseline (SD), -0.63 (4.76), Mediterranean – mixed nuts, n=63, mean change from baseline (SD), -0.23 (3.60) cm

Table S5. Effects of self management, diet, and combination interventions on HbA1c in studies including patients with diabetes

Author, year	Baseline N	Baseline HbA1c, mean (SE), %	N at 12 months	HbA1c at 12 months, mean (SE), %	Between-group difference in change from baseline at 12 months, %	Notes
Self Management						
**Clark M, 2004** [5]						
Control						
Self management						No significant interaction between time and intervention for the combination of five laboratory measurements (cholesterol, HDL, LDL, TG, HbA1c)
**Plotnikoff, 2011** [8]						
Diabetes education	49	7.8 (2.0)[a]	49	7.2 (1.6)[a]		
Diabetes education + physical activity supplement	47	7.3 (1.3)[a]	47	7.2 (1.4)[a]	+0.1 (P>0.05) versus diabetes education	Difference in adjusted change from baseline reported
Diet						
**Abraira, 1980** [1]						HbA1c results not reported
**Razquin, 2009**[9]						HbA1c results not reported
Combination						
**Samaras K, 1997** [12]						
Control	13	6.8 (0.6)	13	Change from baseline: 0.86 (0.27)		
Self management + physical activity	13	5.6 (0.3)	13	Change from baseline: 0.86 (0.29)	0	
**Toobert D, 2011**[14b]						
Usual Care	138	8.4 (0.02)	138	7.8 (0.02)		
Self management + diet + physical activity	142	8.4 (0.03)	142	8.4 (0.03)	0.6% versus usual care	No significant interaction between time and intervention in GEE model

[a] Standard deviation reported
[b] HbA1c at 24 months: Usual care, 7.8%; Self management + diet + physical activity, 8.4% (0.6% vs usual care)

Table S6. Effects of self management, diet, physical activity, and combination interventions on blood pressure

**Author, year**	**Baseline N**	**Baseline BP, mean (SD), mmHg**	**N at 12 months**	**BP at 12 months, mean (SD), mmHg**	**Between-group difference in change from baseline at 12 months (95% CI), mmHg**	**BP at 36 months, mean (SD), mmHg**	**Between-group difference in change from baseline at 36 months (95% CI), mmHg**
Self Management							
**Clark, 2004 [5a]**							
**Plotnikoff, 2011 [8]**							
Diabetes education	49	SBP: 134.0 (16.1)	49	SBP: -9.3 (-12.9 to -5.6)[b]			
		DBP: 76.4 (9.3)		DBP: -4.4(-7.2 to -1.6)[b]			
Diabetes education + physical activity supplement	47	SBP: 134.0 (17.6)	47	SBP: -10.9 (-14.7 to -7.1)[b]	-1.6; P>0.05 versus diabetes education		
		DBP: 76.5 (9.5)		DBP: -5.6 (-8.6 to -2.7)[b]	-1.2; P>0.05 versus diabetes education		
Diet							
**Abraira, 1980[1a]**							
**Zazpe, 2008[11a]**							
Physical Activity Interventions							
**Yates, 2010 [15a]**							
**Anderssen, 1995 [2]**							
Control	43	SBP: 128.7 (1.5)[c]	43	SBP: -0.5 (1.7)[c]			
		DBP: 87.0 (1.1)[c]		DBP: -0.7 (1.3)[c]			
Exercise	49	SBP: 132.1 (1.6)[c]	49	SBP: -2.2 (1.1)[c]	-1.7 (P>0.05) versus control		
		DBP: 89.2 (1.1)[c]		DBP: -2.7 (1.0)[c]	-2.0 (P>0.05) versus control		

Combination							
**Toobert, 2011 [14] [a]**							
**Samaras, 1997[12] [a]**							
**Babazono, 2007 [4]**							
Control	41	SBP: 132.0 (17.8)	41	SBP: 123.3 (15.2)			
		DBP: 79.3 (11.8)		DBP: 75.0 (10.2)			
Self management + diet + physical activity	46	SBP: 127.6 (15.7)	46	SBP: 122.1 (16.3)[d]			
		DBP: 78.2 (9.0)		DBP: 74.5 (10.2)[d]	0.6 versus control		
**Stefanick ML, 1998 [13]**							
**Men**							
**All men**	190	SBP: 114.3 (11.4)					
		DBP: 76.1 (7.4)					
Control	46		46	SBP: 0.3 (7.9)[e]			
				DBP: 1.8 (6.1)[e]			
Diet	49		49	SBP: -1.7 (6.4)[e]			
				DBP: -0.3 (5.2)[e]			
Physical activity	47		47	SBP: -0.6 (7.3)[e]			
				DBP: -1.1 (7.1)[e]			
Diet + physical activity	48		48	SBP: -3.0 (6.8)[ef]	P<0.01 versus control		
				DBP: -3.0 (6.6)[eg]	P<0.01 versus control		
**Women**							
**All women**	177	SBP: 115.5 (12.8)					
		DBP: 73.2 (7.4)					
Control	45		45	SBP: -2.4 (7.6)[e]			
				DBP: -0.6 (5.9)[e]			
Diet	46		46	SBP: -3.5 (9.2)[e]			
				DBP: -1.9 (5.0)[e]			
Physical activity	43		43	SBP: -1.1 (8.9)[e]			
				DBP: -1.4 (5.9)[e]			
Diet + physical activity	43		43	SBP: -3.1 (8.4)[eh]			
				DBP: -2.7 (4.6)[ei]			

**Kumanyika SK, 2005** [7]							
Usual Care						SBP: 0.6 (8.5)[e]	
						DBP: -2.4 (7.0)[e]	
Self management + Diet						SBP: -0.7 (9.0)[e]	SBP: -1.35 (P=0.0165) [j]
						DBP: -3.0 (6.5)[e]	DBP: -0.61 (P=0.16)[j]
**Gram B, 2010** [6]							
Control	22	SBP: 152 (18.3)	20	SBP: 145 (5.0)			
		DBP: 89 (10.7)		DBP: 88 (2.0)			
Self management + Nordic walking	22	SBP: 153 (25.1)	21	SBP: 148 (5.0)	SBP: 1.39 (-11.14 to 13.93) versus control		
		DBP: 88 (11.2)		DBP: 84 (2.0)	DBP: -3.95 (-8.73 to 0.83) versus control		
Self management + exercise prescription	24	SBP: 152 (19.4)	24	SBP: 142 (3.0)	SBP: -3.73 (-14.54 to 7.08) versus control		
		DBP: 85 (10)		DBP: 83 (2.2)	DBP: -4.23 (-9.64 to 1.17)		

[a] Blood pressure not reported
[b] Adjusted value at 12 months - baseline
[c] Standard error reported
[d] Statistical significance of between-group difference in SBP and DBP not reported
[e] Change from baseline reported
[f] P=0.13 for overall difference in SBP between groups from analysis of variance
[g] P=0.003 for overall difference in DBP between groups from analysis of variance
[h] P=0.59 for overall difference in SBP between groups from analysis of variance
[i] P=0.33 for overall difference in DBP between groups from analysis of variance
[j] Adjustment for baseline variables: ethnicity, gender, age, BP, BMI, sodium excretion

Table S7. Effects of self management, diet, physical activity, and combination interventions on blood pressure in race-sex subgroups

**Author, year**	**N**	**Baseline BP**	**Change in BP at 36 months**	**Between-group difference in BP change at 36 mo**
**Kumanyika, 2005** [7]				
**White Men**				
Usual care	345	Mean SBP (SD): 127.1 (6.3)<br>Mean DBP (SD): 85.9 (1.9)	Mean change in SBP from baseline (SD): -0.3 (7.8)<br>Mean change in DBP from baseline (SD): -2.7 (7.7)	Reference
Diet	336	Mean SBP (SD): 127.2 (6.5)<br>Mean DBP (SD): 86.2 (2.0)	Mean SBP (SD): -1.3 (8.5)<br>Mean DBP (SD): -3.0 (6.2)	Mean SBP (95% CI): - 1.1 (-2.4 to 0.2)<br>Mean DBP (95% CI): -0.3 (-1.4 to 0.8)
**Black Men**				
Usual care	47	Mean SBP (SD): 126.2 (6.6)<br>Mean DBP (SD): 85.7 (1.9)	Mean change in SBP from baseline (SD): 1.7 (7.4)<br>Mean change in DBP from baseline (SD): -1.9 (7.0)	Reference
Diet	39	Mean SBP (SD): 128.0 (6.1)<br>Mean DBP (SD): 86.2 (2.0)	Mean SBP (SD): 2.2 (10.3)<br>Mean DBP (SD): -0.6 (8.1)	Mean SBP (95% CI): 0.5 (-3.8 to 4.9)<br>Mean DBP (95% CI): 1.4 (-2.2 to 4.9)
**White Women**				
Usual care	129	Mean SBP (SD): 127.8 (6.5)<br>Mean DBP (SD): 85.7 (1.9)	Mean change in SBP from baseline (SD): 2.1 (10.4)<br>Mean change in DBP from baseline (SD): -1.9 (6.8)	Reference
Diet	129	Mean SBP (SD): 128.2 (6.9)<br>Mean DBP (SD): 85.8 (1.9)	Mean SBP (SD): 0.5 (8.9)<br>Mean DBP (SD): -3.4 (5.8)	Mean SBP (95% CI): -1.5 (-4.0 to 0.9)<br>Mean DBP (95% CI): -1.4 (-3.1 to 0.2)
**Black Women**				
Usual care	56	Mean SBP (SD): 128.0 (6.6)<br>Mean DBP (SD): 85.7 (2.0)	Mean change in SBP from baseline (SD): 2.0 (9.2)<br>Mean change in DBP from baseline (SD): -1.6 (7.5)	Reference
Diet	61	Mean SBP (SD): 129.0 (6.8)<br>Mean DBP (SD): 86.0 (1.8)	Mean SBP (SD): -1.0 (11.1)<br>Mean DBP (SD): -4.0 (8.2)	36 mo<br>Mean SBP (95% CI): -3.0 (-7.2 to 1.3)<br>Mean DBP (95% CI): -2.4 (-5.7 to 0.8)

Table S8. Effects of self management, diet, physical activity, and combination interventions on LDL and HDL cholesterol

**Author, year**	**Baseline N**	**Baseline cholesterol, mean (SD), mg/dl**	**N at 12 months**	**12 months, mean (SD), mg/dl**	**Between-group difference in change from baseline at 12 months (95% CI), mg/dl**
Self Management					
**Clark M, 2004** [5a]					
**Plotnikoff RC, 2011** [8]					
Diabetes education	49	LDL: 112 (1.7)	49	LDL: -15.4 (-27.0 to -7.7)[b]	
		HDL: 46.3 (11.6)		HDL: 3.9 (0.0 to 7.7)[b]	
Diabetes education + physical activity supplement	47	LDL: 104.2 (46.3)	47	LDL: -15.4 (-27.0 to -7.7)[b]	P>0.05 versus diabetes education
		HDL: 42.5 (11.6)		HDL: 3.9 (3.9 to 7.7)[b]	P>0.05 versus diabetes education
Diet					
**Abraira C, 1980** [1c]					
**Zazpe I, 2008** [11c]					
Physical Activity Interventions					
**Yates T, 2010** [15]					
Control	26	HDL: 50.2 [42.5 to 54.0][d]	26	HDL: 0.0 (-3.9 to 3.9)[e]	
Exercise	24	HDL: 50.2 [42.5 to 57.9]	24	HDL: 0.0 (-30.9 to 30.9)	HDL: -0.0 (-3.9 to 3.9); P= 0.808
Exercise + pedometer	24	HDL: 46.3 [42.5 to 54.0]	24	HDL: 0.0 (-3.9 to 3.9)	HDL: -0.0 (-3.9 to 3.9); P=0.569
**Anderssen, 1995** [2]					
Control	43	LDL: 176.4 (5.0)[f]	43	LDL: -8.5 (3.5)[f]	
		HDL: 40.2 (1.2)[f]		HDL: 0.6 (0.6)[f]	
Exercise	49	LDL: 162.9 (3.9)[f]	49	LDL: -5.0 (2.7)[f]	P>0.05 versus control
		HDL: 39.0 (0.8)[f]		HDL: 1.5 (0.8)[f]	P>0.05 versus control
Combination					
**Toobert, 2011** [14c]					
**Samaras K, 1997** [12]					

Control	13	HDL: 42.5 (3.9)[f]	13	HDL: -2.7 (1.5)[hf]	
Self management + physical activity	13	HDL: 42.5(3.9)[f]	13	HDL: -0.4 (1.5)[hf]	
**Babazono A, 2007** [4]					
Control	41	LDL: 123.8. (28.2)	41	LDL: 123.9 (26.6)	
		HDL: 55.7 (12.9)		HDL: 56.5 (15.6)	
Self management + diet + physical activity	46	LDL: 121.0 (29.2)	46	LDL: 119.6 (28.0)[g]	
		HDL: 54.5 (13.4)		HDL: 56.7 (14.2)[g]	
**Stefanick ML, 1998** [13]					
**Men**					
**All men**	190	LDL: 155.8 (14.2)			
		HDL: 35.8 (4.4)			
Control	46		46	LDL: -4.6 (21.1)[h]	
				HDL: -0.2 (4.3)[h]	
Diet	49		49	LDL: -10.8 (18.8)[h]	
				HDL: -0.8 (4.4)[h]	
Physical activity	47		47	LDL: -3.6 (18.8)[h]	
				HDL: 1.2 (4.4)[h]	
Diet + physical activity	48		48	LDL: -20.0 (17.3)[hi]	P<0.001 versus control and physical activity
				HDL: 0.4 (5.3)[hj]	
**Women**					
**All women**	177	LDL: 160.7 (18.4)			
		HDL: 47.0 (6.7)			
Control	45		45	HDL: -2.5 (16.6)[h]	
				LDL: 1.0 (6.1)[h]	
Diet	46		46	LDL: -7.3 (18.9)[h]	
				HDL: 0.3 (6.0)[h]	
Physical activity	43		43	LDL: -5.6 (19.4)[h]	
				HDL: 2.3 (6.7)[h]	
Diet + physical activity	43		43	LDL: -14.5 (22.2)[hk]	P<0.05 versus control
				HDL: -1.1 (6.4)[hl]	
**Kumanyika SK, 2005** [7c]					

**Gram B, 2010** [6]					
Control	22	LDL: 98.8 (25.87)	20	LDL: 100.4 (19.3)[f]	
		HDL: 42.5 (14.3)		HDL: 92.7 (7.7)[f]	
Self management + Nordic walking	22	LDL: 98.8 (32.8)	21	LDL: 69.5 (7.7)[f]	LDL: -15.8 (-39.4 to 7.7) versus control[m]
		HDL: 42.5 (11.2)		HDL: 88.8 (7.7)[f]	HDL: -6.2 (-22.4 to 10.4) versus control
Self management + exercise prescription	24	LDL: 99.2 (23.6)	24	LDL: 100.4 (15.4)[f]	LDL: 12.0 (-23.2 to 46.7) versus control
		HDL: 44.8 (16.6)		HDL: 104.2 (7.7)[f]	HDL: 7.3 (-5.0 to 26.6) versus control

[a] No significant interaction between time and intervention for the combination of five laboratory measurements (cholesterol, HDL, LDL, TG, HbA1c)
[b] Adjusted value at 12 months - baseline
[c] LDL and HDL cholesterol not reported
[d] Median [interquartile range]
[e] Mean change from baseline (95% confidence interval)
[f] Standard error reported
[g] Statistical significance of between-group difference in SBP and DBP not reported
[h] Change from baseline reported
[i] P<0.001 for overall difference in LDL between groups from analysis of variance
[j] P=0.21 for overall difference in HDL between groups from analysis of variance
[k] P=0.03 for overall difference in LDL between groups from analysis of variance
[l] P=0.09 for overall difference in HDL between groups from analysis of variance
[m] Adjusted for baseline value of LDL or HDL

References

1. Abraira C, de Bartolo M, Myscofski JW. Comparison of unmeasured versus exchange diabetic diets in lean adults. Body weight and feeding patterns in a 2-year prospective pilot study. *Am J Clin Nutr.* May 1980;33(5):1064-1070.
2. Anderssen SA, Haaland, A., Hjermann, I., Urdal, P., Gjesdal, K., Holme, I. Oslo Diet and Exercise Study: a one-year randomized intervention trial. Effect on hemostatic variables and other coronary risk factors. *Nutr Metab Cardiovasc Dis.* 1995;5:189-200.
3. Torjesen PA, Birkeland KI, Anderssen SA, Hjermann I, Holme I, Urdal P. Lifestyle changes may reverse development of the insulin resistance syndrome. The Oslo Diet and Exercise Study: a randomized trial. *Diabetes care.* Jan 1997;20(1):26-31.
4. Babazono A, Kame, C., Ishihara, R., Yamamoto, E., Hillman, A.L. Patient-motivated prevention of lifestyle-related disease in Japan. *Dis Manage Health Outcomes.* 2007;15(2):119-126.
5. Clark M, Hampson SE, Avery L, Simpson R. Effects of a tailored lifestyle self-management intervention in patients with type 2 diabetes. *Br J Health Psychol.* Sep 2004;9(Pt 3):365-379.
6. Gram B, Christensen R, Christiansen C, Gram J. Effects of nordic walking and exercise in type 2 diabetes mellitus: a randomized controlled trial. *Clin J Sport Med.* Sep 2010;20(5):355-361.
7. Kumanyika SK, Cook NR, Cutler JA, et al. Sodium reduction for hypertension prevention in overweight adults: further results from the Trials of Hypertension Prevention Phase II. *J Hum Hypertens.* Jan 2005;19(1):33-45.
8. Plotnikoff RC, Pickering MA, Glenn N, et al. The effects of a supplemental, theory-based physical activity counseling intervention for adults with type 2 diabetes. *J Phys Act Health.* Sep 2011;8(7):944-954.
9. Razquin C, Martinez JA, Martinez-Gonzalez MA, Mitjavila MT, Estruch R, Marti A. A 3 years follow-up of a Mediterranean diet rich in virgin olive oil is associated with high plasma antioxidant capacity and reduced body weight gain. *Eur J Clin Nutr.* Dec 2009;63(12):1387-1393.
10. Razquin C, Martinez JA, Martinez-Gonzalez MA, Fernandez-Crehuet J, Santos JM, Marti A. A Mediterranean diet rich in virgin olive oil may reverse the effects of the -174G/C IL6 gene variant on 3-year body weight change. *Mol Nutr Food Res.* May 2010;54 Suppl 1:S75-82.
11. Zazpe I, Sanchez-Tainta A, Estruch R, et al. A large randomized individual and group intervention conducted by registered dietitians increased adherence to Mediterranean-type diets: the PREDIMED study. *J Am Diet Assoc.* Jul 2008;108(7):1134-1144; discussion 1145.
12. Samaras K, Ashwell S, Mackintosh AM, Fleury AC, Campbell LV, Chisholm DJ. Will older sedentary people with non-insulin-dependent diabetes mellitus start exercising? A health promotion model. *Diabetes Res Clin Pract.* Aug 1997;37(2):121-128.
13. Stefanick ML, Mackey S, Sheehan M, Ellsworth N, Haskell WL, Wood PD. Effects of diet and exercise in men and postmenopausal women with low levels of HDL cholesterol and high levels of LDL cholesterol. *N Engl J Med.* Jul 2 1998;339(1):12-20.
14. Toobert DJ, Strycker LA, King DK, Barrera M, Jr., Osuna D, Glasgow RE. Long-term outcomes from a multiple-risk-factor diabetes trial for Latinas: inverted exclamation markViva Bien! *Transl Behav Med.* Sep 2011;1(3):416-426.
15. Yates T, Davies MJ, Gorely T, et al. The effect of increased ambulatory activity on markers of chronic low-grade inflammation: evidence from the PREPARE programme randomized controlled trial. *Diabet Med.* Nov 2010;27(11):1256-1263.
16. Aggarwal A, Freund K, Sato A, et al. Are depressive symptoms associated with cancer screening and cancer stage at diagnosis among postmenopausal women? The Women's Health Initiative Observational Cohort. *Journal of Women's Health (15409996).* 2008;17(8):1353-1361.

17. The Diabetes Prevention Program. Design and methods for a clinical trial in the prevention of type 2 diabetes. *Diabetes care.* Apr 1999;22(4):623-634.
